# The Regulatory Effects of Licochalcone A on the Intestinal Epithelium and Gut Microbiota in Murine Colitis

**DOI:** 10.3390/molecules26144149

**Published:** 2021-07-08

**Authors:** Juan Zhang, Li Cao, Yu Sun, De-Gang Qing, Xiao-Qin Xu, Jun-Chi Wang, Jian-Yong Si, Ning Li

**Affiliations:** 1 Institute of Medicinal Plant Development, Chinese Academy of Medical Sciences and Peking Union Medical College, Beijing 100193, China; ezhangjuane76@sina.com (J.Z.); lcao@implad.ac.cn (L.C.); jcwang@implad.ac.cn (J.-C.W.); 2 Xinjiang Institute of Chinese Materia Medica and Ethnodrug, Ürümqi 830002, China; sunyu6541715@126.com (Y.S.); qingdegang@sina.com (D.-G.Q.); xjxuxq@163.com (X.-Q.X.); 3 School of Traditional Chinese Materia Medica, Shenyang Pharmaceutical University, Shenyang 110016, China

**Keywords:** Licochalcone A, ulcerative colitis, gut barrier, microbiota, MAPK

## Abstract

The gut epithelium is a mechanical barrier that protects the host from the luminal microenvironment and interacts with the gut microflora, which influences the development and progression of ulcerative colitis (UC). Licochalcone A (LA) exerts anti-inflammatory effects against UC; however, whether it also regulates both the gut barrier and microbiota during colitis is unknown. The current study was conducted to reveal the regulatory effects of LA on the intestinal epithelium and gut microflora in C57BL/6 mice subjected to dextran sodium sulfate (DSS). Sulfasalazine (SASP) was used as the positive control. Results of clinical symptoms evaluation, hematoxylin, and eosin (H&E) staining, and enzyme-linked immunosorbent (ELISA) assays showed that LA significantly inhibited DSS-induced weight loss, disease activity index (DAI) increase, histological damage, and gut inflammation. Additionally, terminal deoxynucleotidyl transferase dUTP nick end labeling (TUNEL) and immunohistochemical (IHC) analysis showed that LA maintained the integrity of the intestinal barrier by suppressing cell apoptosis and preserving the expression of tight junction (TJ) proteins. Notably, the optimal dose of LA for gut barrier preservation was low, while that for anti-inflammatory effects was high, indicating that LA might preserve gut barrier integrity via direct effects on the epithelial cells (ECs) and TJ proteins. Furthermore, 16S rRNA analysis suggested that the regulatory effect of LA on the gut microbiota differed distinctly according to dose. Correlation analysis indicated that a low dose of LA significantly modulated the intestinal barrier-associated bacteria as compared with a moderate or high dose of LA. Western blot (WB) analysis indicated that LA exhibited anti-UC activity partly by blocking the mitogen-activated protein kinase (MAPK) pathway. Our results further elucidate the pharmacological activity of LA against UC and will provide valuable information for future studies regarding on the regulatory effects of LA on enteric diseases.

## 1. Introduction

As a major inflammatory disease, ulcerative colitis (UC) has been gaining increasing attention worldwide in recent years [1]. Despite the efficacy of several types of anti-UC drugs, there still exists an unsatisfactory curative effect, high recurrence, and potential side effects, including allergic reactions, anti-antibody reactions, and infection [2,3,4,5]. Therefore, it is of major importance and urgency to discover novel anti-UC drugs with higher safety and efficacy rates.

It is known that UC is characterized by colonic and rectal injury, including ulceration of the mucosa and serious inflammation [6]. Since the cause of UC is not entirely clear, anti-inflammatory drugs are commonly used to relieve the disease’s symptoms. However, UC is thought to be caused by an intricate interplay of host genetics, host immune system, the gut barrier, the environment, and the gut microbiota. Thus, novel drugs that treat or target other aspects of the disease are needed. Among these multiple factors involved in UC, gut barrier damage has been identified as a vital etiological factor in colitis [7,8]. The intestinal barrier—the first mechanical defense against pathogens and antigens—is formed by tight junctions (TJs), adhesive junctions, and desmosomes between epithelial cells (ECs) [9]. A growing number of studies strongly support that the epithelial barrier plays a vital role in regulating gut homeostasis and also interacts with microbes to influence colitis. Once the gut barrier is disrupted, which is represented by changes in TJ proteins that include claudin-1, occludin, and ZO-1, and an increase in EC apoptosis [8,10], luminal antigens and toxins are able to penetrate the underlying tissue, and the consequent exposure thereof to the luminal antigens induces an inflammatory response and the release of inflammatory cytokines, which trigger further degradation of TJs [11]. Additionally, the gut barrier is intimately influenced by the colonic microflora. The intestinal microbiota maintains homeostasis of the gut barrier by balancing the proliferation and apoptosis of ECs, protecting TJs, and intensifying the mucus layer [4]. Changes in the composition and function of the gut microbiota cause alterations in intestinal ECs and TJ proteins [12,13,14], both of which are responsible for controlling gut permeability. An increase in gut permeability is known to facilitate microbial translocation, perpetuating UC [15]. Therefore, exploiting drugs that synergize gut barrier protection and microbiota regulation could be a promising avenue for UC treatment.

In recent years, researchers have taken interests in the regulatory effects of herbally derived ingredients on the intestinal epithelium and gut microbiota [16]. Several components have been reported to exhibit desirable effects on both [17,18]. Licochalcone A (LA) is the dominant characteristic chalcone in *Glycyrrhiza inflata* [19]. We previously demonstrated that LA can relieve UC symptoms in mice via anti-inflammatory and anti-oxidative effects [20]. However, whether LA plays an antagonistic role in the intestinal barrier and microflora in UC is not clear. Therefore, we further explored the pharmacological activity of LA in dextran sulfate sodium (DSS)-induced UC.

## 2. Results

### 2.1. LA Protected against DSS-Induced UC

**Figure 1:**
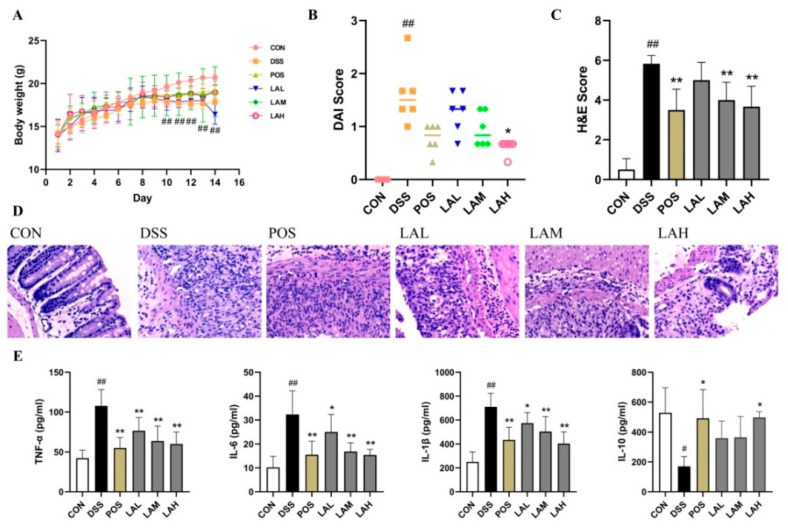
LA protected mice against dextran sodium sulfate (DSS)-induced ulcerative colitis (UC) and attenuated colonic inflammation. (**A**) Body weight of mice over the course of 14 days. (**B**) Disease activity index (DAI) score of each mouse on day 14. (**C**) Hematoxylin and eosin (H&E) scores. (**D**) Photomicrographs of H&E staining (200×). (**E**) Levels of TNF-α, IL-6, IL-1β, and IL-10 in colon tissue. Data are expressed as mean ±SD (*n* = 6); # *p* < 0.05 and ## *p* < 0.01 vs. CON, * *p* < 0.05 and ** *p* < 0.01 vs. DSS.

To evaluate the action of LA on colitis, the clinical symptoms of DSS-induced UC were firstly determined. The body weight of healthy control mice (CON group) showed a steady increase over the course of the 14 days, while that of DSS-subjected mice increased to a lesser extent throughout the first 9 days but began to decline at day 10. There was a significant difference between the DSS and CON groups from days 10 to 14 (*p* < 0.01, Figure 1A). However, sulfasalazine (SASP) and moderate- and high-dose LA mildly alleviated body weight loss (*p* > 0.05). Compared with the disease activity index (DAI) score of CON mice (0.00 ± 0.00), DSS administration increased DAI score (1.61 ± 0.58) due to body weight loss, diarrhea, and bleeding (*p* < 0.01, Figure 1B). However, LA treatment reduced DAI score in a dose-dependent manner, significantly so in the high-dose (LAH) group (0.61 ± 0.14) (*p* < 0.05). Hematoxylin and eosin (H&E) staining showed that DSS effectively induced ulceration, inflammatory-cell infiltration, and crypt atrophy (Figure 1D). As expected, LA administration inhibited histological injury. The H&E score of the DSS group was significantly increased over that of the CON group (*p* < 0.01, Figure 1C). In contrast, SASP and moderate- and high-dose LA administration significantly decreased H&E score (*p* < 0.01). Collectively, these results demonstrated that LA ameliorated DSS-induced murine colitis.

### 2.2. LA Suppressed Colonic Inflammation

Aggravation of inflammatory reactions, represented by the improper release of inflammatory cytokines, is a hallmark of DSS-induced colitis [21]. Therefore, the cytokine levels in colonic tissue were assessed by using enzyme-linked immunosorbent (ELISA) assay to identify the anti-inflammatory effect of LA. Levels of TNF-α, IL-6, and IL-1β were significantly increased in DSS-treated mice compared with CON mice (*p* < 0.01, Figure 1E), while that of IL-10 was significantly decreased (*p* < 0.05) in DSS-subjected mice. Consistent with the therapeutic action of LA on clinical symptoms, LA dose-dependently inhibited the aberrant release of pro- and anti-inflammatory cytokines. In particular, both SASP and high-dose LA significantly downregulated levels of TNF-α, IL-6, and IL-1β (*p* < 0.01), while upregulating that of IL-10 (*p* < 0.05) in the treatment group *versus* the DSS group. These results implied that LA administration ameliorated murine colitis by inhibiting colonic inflammation.

### 2.3. LA Preserved the Gut Barrier Integrity

**Figure 2:**
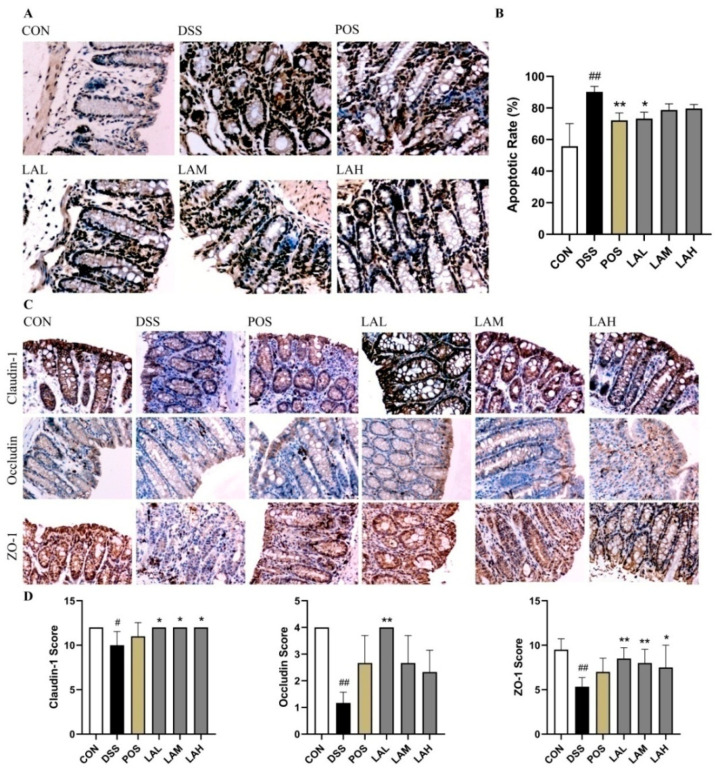
LA preserved the gut barrier integrity in UC mice. (**A**) TUNEL assay images (200×). (**B**) Rate of epithelial cell (EC) apoptosis. (**C**) Immunohistochemistry images (200×). (**D**) Claudin-1, occludin, and ZO-1 scores. Data are expressed as mean ± SD (*n* = 6); # *p* < 0.05 and ## *p* < 0.01 vs. CON, * *p* < 0.05 and ** *p* < 0.01 vs. DSS.

Increased EC apoptosis [22,23] and improper TJ protein expression [24] in UC can induce gut barrier injury, which exposures the intestinal epithelium to opportunistic pathogens that can trigger continuous inflammation [25]. Therefore, terminal deoxynucleotidyl transferase dUTP nick end labeling (TUNEL) and immunohistochemical (IHC) assays were performed on the colonic tissue of CON and DSS-subjected mice in order to define the protective effects of LA on the gut barrier. As expected, DSS treatment greatly increased EC apoptosis (Figure 2A) and decreased the expression of TJ proteins (Figure 2C), while LA effectively suppressed these changes. DSS exposure induced a significant increase in the apoptotic rate of ECs as compared with the CON group (*p* < 0.01, Figure 2B). However, both SASP and low-dose LA significantly suppressed cell apoptosis (*p* < 0.01, and *p* < 0.05, respectively, Figure 2B). Additionally, expression levels of claudin-1, occludin, and ZO-1 were significantly decreased in DSS mice (*p* < 0.05, *p* < 0.01, and *p* < 0.01, respectively; Figure 2D), indicating compromised TJ integrity. In contrast, all three doses of LA protected DSS-treated mice from TJ damage. Notably, low-dose LA showed the most significant protective effect on the expression of claudin-1, occludin, and ZO-1 (*p* < 0.05, *p* < 0.01, and *p* < 0.01, respectively).

### 2.4. LA Reshaped the Intestinal Microbiota

**Figure 3:**
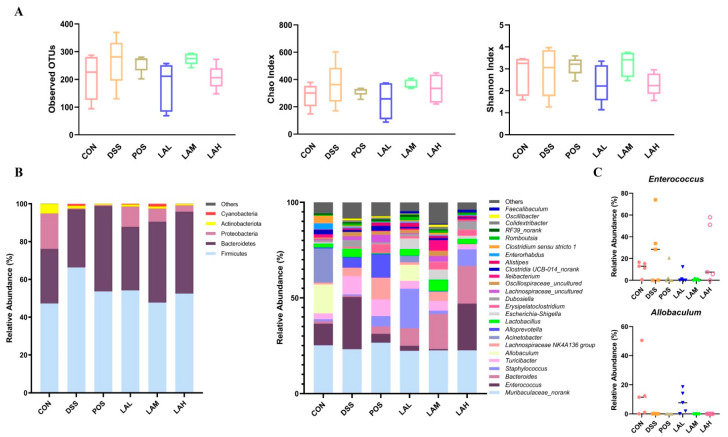
LA regulated the intestinal flora in DSS-subjected mice. (**A**) Observed OTUs, Chao index, and Shannon index. (**B**) Gut bacterial distribution at the phylum and genus levels. (**C**) Relative abundances of *Enterococcus* and *Allobaculum*. Data are expressed as mean ± SD (*n* = 5).

To investigate the correlation between gut flora and the action of LA administration during murine colitis, we tested fecal bacteria using 16S rRNA analysis. The detected operational taxonomic units (OTUs), Chao index, and Shannon index of the DSS group were increased, but not a statistically different degree compared with the CON group (*p* > 0.05, Figure 3A). Low-dose LA mildly inhibited the changes in the observed OTUs, Chao index and Shannon index (*p* > 0.05). Taxonomic profiling demonstrated that the dominant phyla in the CON group were Firmicutes, Bacteroidetes, Proteobacteria, and Actinobacteria (Figure 3B). Administration of DSS shifted bacterial composition by increasing the proportions of Firmicutes and Bacteroidetes and decreasing that of Proteobacteria, which was consistent with alteration of the luminal microbiota in UC mice [9]. However, both SASP and LA downregulated the level of Firmicutes, while they upregulating that of Bacteroidetes. At the genus level, bacterial compositions of six groups were differed with each other. Compared to the CON group, increased *Enterococcus* and *Turicibacter,* and decreased *Allobaculum* and *Acinetobacter* were observed in UC mice. Low-dose LA exhibited regulatory effects on inhibiting *Enterococcus* and promoting *Allobaculum* (Figure 3C), indicating that LA might have an effect on modulating bacterial structure in colitis mice.

**Figure 4:**
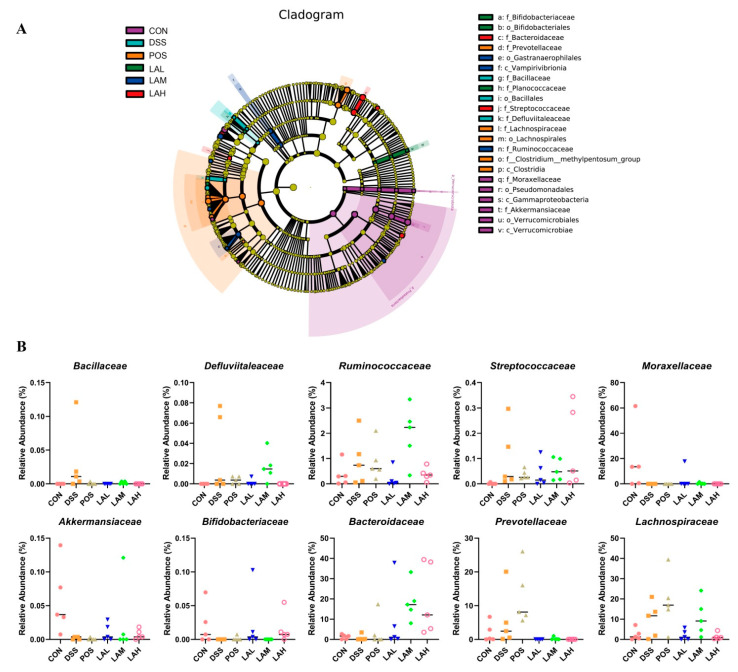
Biomarkers in six groups. (**A**) Cladogram of linear discriminant analysis effect size (LEfSe) comparison analysis. (**B**) Relative abundances of families that were significantly modulated by LA and SASP. Data are expressed as mean ± SD (*n* = 5).

Linear discriminant analysis effect size (LEfSe) analysis was carried out to discover biomarkers in each group. The results showed that *Bacillaceae, Bacillales*, and *Defluviitaleaceae* were biomarkers in the DSS group, while *Moraxellaceae*, *Pseudomonadales*, *Akkermansiaceae*, and *Verrucomicrobiales* were biomarkers in the CON group (Figure 4A). The regulatory effects of LA on gut microbiota in DSS-exposed mice distinctly varied by dose. *Bifidobacteriaceae*, *Bifidobacteriales*, and *Planococcaceae* differed in LAL-treated mice; *Gastranaerophilales*, *Vampirivibrionia*, and *Ruminococcaceae* differed in mice treated with a moderate dose of LA; and *Bacteroidaceae* and *Streptococcaceae* differed in LAH-treated mice. Altogether, the relative abundances of *Bacillaceae*, *Defluviitaleaceae*, *Ruminococcaceae*, and *Streptococcaceae* were increased, while that of *Moraxellaceae, Akkermansiaceae, and Bifidobacteriales were decreased* in DSS-subjected mice, then adjusted to the control level after treatment of LA (Figure 4B). However, only low-dose of LA modulated all the above changes due to DSS administration. Interestingly, three doses of LA especially at a high-dose promoted the growth of *Bacteroidaceae*, even though DSS did not regulate on it. Additionally, biomarkers in SASP-treated mice were *Prevotellaceae* and *Lachnospiraceae*, which were upregulated by DSS. The regulatory effects of SASP on the two families were further boosting, which is completely opposite to that of LA. These data suggested that the regulatory effects of SASP and LA on gut microflora composition in colitis mice were different due to their different mechanisms, and LA could regulate the intestinal microflora structure more closely to the CON group compared with SASP.

**Figure 5:**
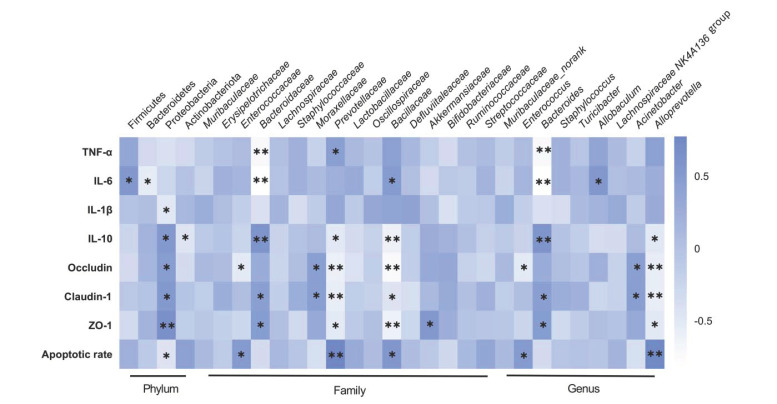
Correlation analysis heatmap (only significant correlations (* *p* < 0.05, ** *p* < 0.01) are presented).

Correlation analysis was performed based on the experimental data to identify a correlation among bacteria, inflammatory cytokines, TJ proteins, and rate of apoptosis in DSS-treated mice [8]. The data indicated a notable correlation between the proportion of bacteria and other parameters (Figure 5). For example, at the phylum level, Firmicutes was positively associated (*p* < 0.05) and Bacteroidetes was negatively associated with IL-6 (*p* < 0.05). At the family level, *Prevotellaceae* showed positive correlation with TNF-α (*p* < 0.05) and apoptotic rate (*p* < 0.01), but negative correlation with IL-10 (*p* < 0.05), occludin (*p* < 0.01), claudin-1 (*p* < 0.01) and ZO-1 (*p* < 0.05). *Bacillaceae* showed positive correlation with IL-6 (*p* < 0.05) and apoptotic rate (*p* < 0.05), but negative correlation with IL-10 (*p* < 0.01), occludin (*p* < 0.01), claudin-1 (*p* < 0.05) and ZO-1 (*p*< 0.01). Similar results were noted in *Moraxellaceae* and *Akkermansiaceae*. Altogether, these data suggested that LA might protect mice from UC by inhibiting harmful bacteria, and boosting beneficial bacteria. Interestingly, we found that *Bacteroidaceae* demonstrated notable negative correlation with TNF-α (*p* < 0.01) and IL-6 (*p* < 0.01), but positive correlation with IL-10 (*p* < 0.01), claudin-1 (*p* < 0.05) and ZO-1 (*p* < 0.05). These results suggested that the effects of LA on promoting *Bacteroidaceae* were beneficial to relieve colitis. Above all, the results implied that the effects of LA on murine colitis were synergistic actions of anti-inflammatory, gut barrier, and bacterial regulatory activities.

### 2.5. LA Blocked the Mitogen-Activated Protein Kinase (MAPK) Pathway

**Figure 6:**
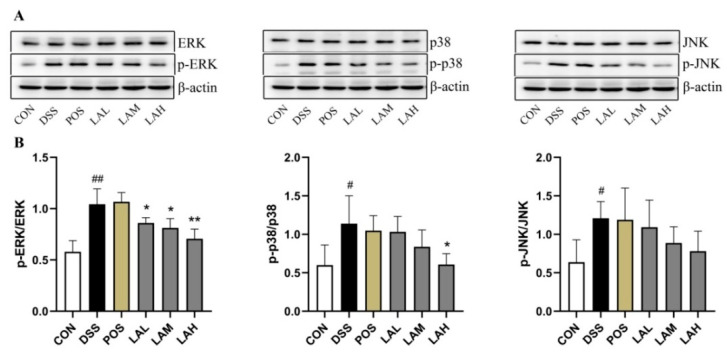
LA inhibited expression of MAPK pathway-related proteins. (**A**) Western blot (WB) images. (**B**) Relative levels of p-ERK/ERK, p-p38/p38, and p-JNK/JNK. Data are expressed as mean ± SD (*n* = 3), # *p* < 0.05 and ## *p* < 0.01 vs. CON, * *p* < 0.05 and ** *p* < 0.01 vs. DSS.

Improper activation of the MAPK pathway, which results in phosphorylation of p38, ERK, and JNK, is frequently observed in patients with UC and in mice subjected to experimental colitis [26,27]. Additionally, activation of this pathway has been shown to exacerbate inflammatory reactions and impair the gut barrier [23]. In the current study, tissue levels of MAPK pathway-related proteins were assessed via Western blot (WB) in order to further reveal the mechanisms of LA in murine colitis. While DSS exposure significantly increased the levels of p-ERK, p-p38, and p-JNK (*p* < 0.01, *p* < 0.05, and *p* < 0.05, respectively, Figure 6A,B), LA administration dose-dependently decreased the levels of all three phosphorylated proteins. These data indicated that LA could block the MAPK pathway, which in turn dampened colonic inflammation.

## 3. Discussion

In the present study, LA significantly ameliorated DSS-induced clinical symptoms and release of pro- and anti-inflammatory factors, indicating that LA can relieve murine colitis at least in part via anti-inflammation. LA also preserved the integrity of the colonic barrier by inhibiting cell apoptosis and protecting the expression of TJ proteins. Interestingly, the protective effect of LA on the gut barrier of UC mice was negatively correlated with dose, which was inconsistent with its anti-inflammatory effect. One possible reason for this discrepancy is that LA might preserve gut barrier integrity via direct effects on intestinal ECs and TJ proteins, regardless of its anti-inflammatory action.

We also determined whether the anti-inflammatory and gut barrier-protective effects of LA on colitis correlated with the composition of intestinal bacteria. Results of 16S rRNA analysis showed that DSS mildly increased the bacterial richness and diversity in mice, which was consistent with a previous study [28]. We therefore analyzed the microbial distribution. As previously reported, DSS boosted the genera *Enterococcus* [29], *Turicibacter* [30], *Lactobacillus* [15], *Dubosiella* [15], *and Alloprevotella* [31,32], most of which are also considered harmful bacteria in colitis [15,33,34]. These results suggested that the main contribution of DSS toward increasing bacterial richness and diversity was harmful bacteria. LA suppressed DSS induced changes, evidenced by the suppressive effects of low- and moderate-dose LA on the increased *Enterococcus* due to DSS. *Enterococcus* is pathogenic and has been shown to be negatively correlated with human health; many species belonging to this genus can induce colitis [33,35]. In our study, *Enterococcus* was the most abundant genus in DSS-subjected mice. Correlation analysis showed that *Enterococcus* negatively correlated with TJ proteins and positively correlated with apoptotic rate of intestinal ECs, indicating that it was not conducive to preserving gut barrier integrity. LEfSe analysis further revealed the action of LA on gut microflora, which implied that *Bacillaceae*, *Defluviitaleaceae*, *Ruminococcaceae*, *Streptococcaceae*, *Moraxellaceae*, *Akkermansiaceae*, and *Bifidobacteriales* were involved in the therapeutic effects of LA against murine colitis. Correlation analysis suggested that LA especially at a low-dose could alleviate colitis through enhancing the relative abundance of gut barrier-beneficial bacteria such as *Akkermansiaceae*, and decreasing the levels of gut barrier-harmful bacteria including *Prevotellaceae* and *Bacillaceae*. *Akkermansiaceae* is beneficial bacteria [36], and it is reported that decreased *Akkermansiaceae* due to DSS can be promoted by Jangkanghwan, which exhibited inhibitory effect on murine colitis [37]. Specifically, strains belonging to *Akkermansiaceae* such as *Akkermansia muciniphila*, can improve the gut barrier function [37]. *Prevotellaceae* is one of the representative taxa in UC patients [38]. Study conducted by Wright et al. demonstrated that *Prevotellaceae* can destroy the mucosal barrier function via sulfatases production [39]. Taken together, the results suggested that LA might ameliorate UC through adjusting intestinal barrier-related bacteria. Furthermore, low-dose LA inhibited the decreased *Allobaculum* induced by DSS, which was consistent with a previous report [40]. However, our correlation analysis showed that *Allobaculum* was positively correlated with IL-6. Therefore, it is reasonable to speculate that low-dose LA had a better regulatory effect on gut barrier-related bacteria than inflammation-associated bacteria. This finding explained, to some extent, the inconsistency between the doses required for the anti-inflammatory and gut barrier-protective effects of LA. Even though the effects of some strains such as *Enterococcus* on the gut barrier have not been completely ascertained, our conclusion is partially supported by a previous study [41], which documented that stimulating Caco-2 cells with lipopolysaccharide (LPS) derived from five different Gram-negative bacteria resulted in different cell characteristics. *Serratia marcescens* significantly promoted TNF-α expression, while *Klebsiella pneumoniae* induced substantially more IL-10. However, *Serratia marcescens* and *Escherichia coli* showed the greatest effect on monolayer permeability, even though the pro-inflammatory effect of *Escherichia coli* was not prominent.

Given the regulatory effect of LA on gut inflammation and intestinal epithelium in UC, we further investigated the inflammation- and gut barrier-related pathways involved in colitis. The MAPK pathway is highly activated during the development of colitis [42,43] and plays not only an anti-inflammatory role in the intestine [44], but also the important roles of regulating the expression of TJ proteins [45] and cell apoptosis [46]. Accumulating evidence has demonstrated that bioactive ingredients derived from natural products can suppress inflammatory responses and rebuild the gut barrier via the MAPK pathway. For example, via this pathway, chlorogenic acid attenuates DSS-induced colitis in mice by inhibiting inflammation and cell apoptosis [47]. Administration of LP-5, a peptide derived from a chicken by-product, can inhibit DSS-induced phosphorylation of JNK and p38 to increase levels of TJ proteins and suppress inflammatory reaction, thereby alleviating colitis [45]. Our results implied that the MAPK pathway was involved in the ameliorative action of LA against colitis. However, it is worth noting that the blocking effect of LA on MAPK-related proteins increased as the concentration of LA increased, which was inconsistent with the gut barrier-beneficial effect. We therefore hypothesized that the major outcome of LA-dependent inhibition of the MAPK pathway was a decrease in inflammation rather than a protective effect on the gut barrier. Detailed studies are required to understand the underlying molecular mechanisms of LA-dependent gut barrier preservation.

We had previously demonstrated that LA’s ameliorative effects against DSS-induced UC were through anti-inflammation [20]. The results of the current study revealed another new mechanism by which LA sequentially regulated gut inflammation, the intestinal microbiota and gut barrier during DSS-induced UC. Our exploration of the pharmacological activity of LA will provide valuable clues for further study of its regulatory effects on enteric diseases.

## 4. Materials and Methods

### 4.1. Chemicals and Animals

DSS was obtained from MP Biomedicals (Solon, OH, USA). We purchased SASP from Sigma-Aldrich (St. Louis, MO, USA). Mouse TNF-α, IL-6, IL-1β, and IL-10 ELISA kits were obtained from Multi Sciences Biotech (Hangzhou, China). The TUNEL assay kit, and the anti-ZO-1 antibody and anti-Claudin-1 antibodies were from Boster Biological Tech (Wuhan, China). We purchased 3,3′-diaminobenzidine (DAB), anti-Occludin antibody, dibutylphthalate polystyrene xylene (DPX) medium, and SDS-PAGE gels from MX Biotech (Xiamen, China), Bioss (Beijing, China), Zhongshan Bio. (Beijing, China) and Sangon Biotech (Germany), respectively. ERK1/2, p-ERK1/2, p38,p-p38, JNK, and p-JNK antibodies were obtained from Abcam (Cambridge, UK). We purchased the chemiluminescence substrate from Thermo Fisher Scientific (Waltham, MA, USA). Male C57BL/6 mice were purchased from the Animal Experiment Center of Xinjiang Medical University (XMU; Ürümqi, China).

### 4.2. DSS-Induced Colitis

Mice (19 ± 1 g; age, 6–7 weeks) were housed in a room on a 12-h light/dark cycle and under controlled conditions (22 ± 3 °C, 60–80% relative humidity). We induced UC by adding 1.5% *w*/*v* DSS (36–50 kDa) to the mice’s drinking water for 14 days. On days 1 to 14, normal control mice (CON), DSS model mice (DSS), SASP-treated mice (POS, 10 mg/kg/day), and LA-treated mice (LAL, 20 mg/kg/day; LAM, 40 mg/kg/day; LAH, 80 mg/kg/day) drank from separate water bowls.

### 4.3. Assessment of Disease Activity

According to Cooper scoring criteria [11], DAI scores were assigned based on the evaluation of the body weight loss, stool consistency, and fecal occult blood.

### 4.4. Evaluation of Histology

Colon segments were fixed in 4% paraformaldehyde, embedded in paraffin, and sectioned into 5-μm-thick slices for H&E staining. DSS-induced histological injury was scored base on epithelium injury and inflammatory-cell infiltration, as outlined previously [42]. Each sample was photographed using a Nikon E200 microscope (Nikon Co. Ltd., Tokyo, Japan).

### 4.5. ELISA Assay

After mouse colon were washed, homogenized, and centrifuged, the levels of TNF-α, IL-6, IL-1β, and IL-10 in the supernatants were examined using ELISA kits per manufacturer’s protocol.

### 4.6. TUNEL Assay

Each sample was photographed under the Nikon E200 microscope (Nikon Co. Ltd., Tokyo, Japan). Apoptosis of colonic cells was measured via TUNEL assay. We dewaxed and hydrated the paraffin-embedded colon slices, performed antigen retrieval using proteinase K, and then incubated the colonic sections with TUNEL assay kit reagents per manufacturer’s protocol. The nuclei were developed with DAB and hematoxylin; blue represents apoptotic cell. A photomicrograph of each sample was obtained with a Nikon E200 microscope.

### 4.7. IHC

For IHC, colonic slices were dewaxed and hydrated. Endogenous peroxidase activity was blocked with 3% H_2_O_2_ solution following antigen retrieval. Sections were incubated with antibodies against ZO-1 (1:500), Claudin1 (1:1000), and Occludin (1:100) at 4 °C overnight. After washing, tissues were incubated at room temperature (RT) for 20 min with horseradish peroxide (HRP)-conjugated secondary antibodies. The sections were then developed with DAB, counterstained using hematoxylin, and mounted with DPX medium. Images were obtained using a Nikon E200 microscope.

### 4.8. 16S rRNA Analysis

The fecal microbial DNA was extracted by using SDS-CTAB method. After the 16S rRNA amplification (v4–v5: 515F-907R) was conducted with PCR (ABI, Vernon, CA, USA) using forward primer 515F-5′-GTGCCAGCMGCCGCGG-3′ and reverse primer 907R-5′- CCGTCAATTCMTTTRAGTTT-3′, PCR products were purified with an AxyPrepDNA gel extraction kit (Axygen, San Diego, CA, USA) and qualified with QuantiFluor™ -ST (Promega, WI, USA). Subsequently, the amplified library was established via NEB Next^®^ Ultra™ DNA Library Prep Kit (Illumina, CA, USA). Finally, 16S rRNA analysis was performed by using Illumina PE250 system and 250bp/300bp paired-end reads were generated. Sequencing data were analyzed by UPARSE software package using the UPARSE-OTU and UPARSE-OUT-REF algorithms. R scripts were used to conduct data analysis, and Lefse was used for the quantitative analysis of biomarkers within different groups [48].

### 4.9. Western Blot

Aliquots of tissue samples containing 20 μg of protein were loaded in 5% SDS-PAGE gel containing β-mercaptoethanol, and then electro-transferred onto PVDF membranes. The membranes were then blocked in 5% non-fat milk and incubated with the following antibodies at 4 °C overnight: ERK1/2 (1:6000), p-ERK1/2 (1:1000), p38 (1:3000), p-p38 (1:1000), JNK (1:800), and p-JNK (1:1500). Subsequently, the membranes were incubated with the corresponding secondary antibodies at RT for 1 h. Protein bands were visualized using a chemiluminescence substrate, and images were obtained using a ChemiScope 3000 (Qinxiang Co. Ltd., Shanghai, China).

### 4.10. Statistical Analysis

Results are presented as mean ±SD. After Kolmogorov–Smirnov tests and homogeneity test of variance, data were subjected to one-way ANOVA or one-way nonparametric ANOVA Kruskal–Wallis test using SPSS 22.0. *p* < 0.05 and *p* < 0.01 were considered significant. GraphPad Prism 8 was used to design the figures.

## Data Availability

All data used to support the findings of this study are available from the corresponding authors upon request.

## Sample Availability

Samples of LA are available from J.Z.
